# Experimental evaluation of a linear angular momentum multiplexed radio link for moving platforms

**DOI:** 10.1098/rspa.2021.0295

**Published:** 2021-10

**Authors:** Ben Allen, Tim W. C. Brown, Timothy D. Drysdale

**Affiliations:** ^1^ Department of Engineering Science, University of Oxford, Parks Road, Oxford OX1 3PJ, UK; ^2^ Network Rail, Elder Gate, Milton Keynes MK9 1ER, UK; ^3^ Institute for Communication, Systems, University of Surrey, Guildford GU2 7XH, UK; ^4^ School of Engineering, University of Edinburgh, Edinburgh EH9 3FB, UK

**Keywords:** wireless communications, linear angular momentum multiplexing, antenna arrays, spectral efficiency, railway communications

## Abstract

Linear angular momentum multiplexing (LAMM) has recently been proposed for high spectral-efficiency communications between moving platforms, such as between trains and ground infrastructure. We present performance results obtained from a scale experimental system comprising a 2 × 2 antenna system operating at 2.35 GHz. The link transmitted two independent video streams, using RF pre-coding and software-defined radios to modulate and up/down-convert the signals. Linear motion is introduced to demonstrate the translation-invariance of the technique. We interpret the measured data with the aid of an analytical model to show that crosstalk between the two channels is at levels low enough to consistently support the video streams without interruption. Specifically, our results show spectral efficiency is consistently higher when LAMM coding is enabled compared with an uncoded channel.

## 1. Introduction

It is a difficult technical challenge to provide train operators and passengers with wireless connectivity, especially at data rates approaching those found in many other settings. Confounding factors are the high density of passengers and the fast train speed with respect to the ground infrastructure. Currently, train-to-ground connectivity usually relies on cellular gateways placed on trains that connect to the base stations, which are at or near to the trackside [1]. For coverage in tunnels, in-fill antennas or leaky feeders are also used [2,3]. Given the limitations on available spectrum resources in mobile bands, and the reduced coverage and spectral efficiency of mobile communication systems outside of urban areas, coverage is patchy or non-existent during a significant proportion of train journeys. An alternative approach is required, which does not suffer from the side effects of optimizations made to suit urban environments, especially given projections for increased data demand on trains as they travel between urban areas.

Insights provided in [4] suggest that by 2025, the average expected data requirements could increase to 3 Mbits s^−1^ per train passenger. This places further pressure on finding a suitable means of connectivity, with the required performance being well beyond that currently provided by cellular technologies. Mobile technologies currently in use often struggle to provide even the estimated unconstrained demand in 2018 of 150 kbits s^−1^ per train passenger [4]. From 2025, a total data rate of 9 Gbits s^−1^ may be required to serve trains having up to 3000 passengers. As well as passenger connectivity, digital operational requirements for the train include on-board sensors [5], closed circuit television links and other auxiliary data links [6]. Trackside base stations could potentially provide this level of service by using millimetre-wave broadband connections, although the inter-site distance is expected to be less than 1 km [7]. Local weather conditions may require even shorter link distances to accommodate atmospheric attenuation [8]. The deployment of the required number of masts is expected to be costly, even at 1 km spacing. Consequently, there is a pressing need for a robust, low-cost, aesthetically acceptable and reliable solution to connect trains to ground telecommunications infrastructure, which has the necessary bandwidth to meet the future requirements. A solution that works for railways could also apply to other fast-moving platforms such as hyperloop, underground railway systems and even large road vehicles for mass passenger transit and autonomous load hauling.

**Figure 1.**
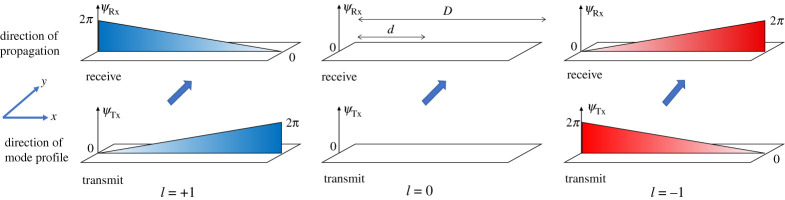
Phase profiles for LAM modes *l* = 0 and *l* = ±1. (Online version in colour.)

Recently, linear angular momentum multiplexing (LAMM) was proposed in [9,10] for providing ultra-spectrally efficient train-to-ground connectivity that is likely to be low-cost, robust and low-profile. Analysis in [9] showed that 9 Gb s^−1^ can be communicated in a 15 MHz bandwidth. LAMM can be thought of as a line-of-sight multiple input/multiple output communication system with carefully chosen fixed pre-coding, i.e. specific phase shifts applied to the transmit and receive antenna elements. LAMM was conceived through research into orbital angular momentum (OAM) radio such as [11–14], where independent electromagnetic field modes are created that can each carry a separate data channel and achieve unprecedented channel capacities for point-to-point radio links [15]. For OAM, the modes exist in a circle around the direction of propagation. By contrast, for LAMM, the modes exist along a line, e.g. along a railway track; and the signals can be picked up by using a linear antenna array positioned along a train. As with any electromagnetic wave, the LAMM field will consist of a spatial amplitude and phase profile along with a polarization vector. For LAMM, it is convenient to consider it as propagating over a short line-of-sight transmit to receive distance as depicted in figure 1 where *D* is the total antenna distance, *d* is the partial distance at a point along the antenna, *ψ*_Tx_ is the phase required at the transmitter and *ψ*_Rx_ the phase required at the receiver. This figure also shows the phase profile of three example LAM modes (with mode numbers *l* = 0 and *l* = ±1) that are discussed in §2. In terms of a railway track, we can consider the train and rails running in a transverse direction to the line-of-sight in either direction as indicated in figure 1. However, note that in practice, all three modes would be co-located, but they are separated here for clarity, where the depicted phase profile of each of the two modes run parallel to the track in the *x*-direction. This results in a non-zero phase gradient in the direction of travel, although the pre-coding remains independent of the offset of the two arrays in the *x-*direction because the phase gradient is invariant. This configuration is the most compact of all possible arrangements because it allows the transmit and receive arrays to be reduced to one dimension, with the long dimension running alongside the track. Any other LAMM configuration requires at least a two-dimensional array, which is beyond the scope of the present paper.

This paper reports an experimental LAMM system that was configured to send two digital video streams over a short distance, representing a track-to-train communications link, scaled to laboratory dimensions. The system is intended to show two video streams successfully received as one antenna array incrementally moves along a path, mimicking the motion of a train-mounted antenna travelling along a track. The system consists of a 2 × 2 antenna system such that two LAM modes are created. Each data stream is allocated its own LAM mode while using the same carrier frequency and polarization and being transmitted at the same time as the other. Software-defined radios (SDRs) are used to transmit and receive the signals, with video decoding done on an associated computer. This demonstration builds on the theoretical analysis of a 2 × 2 LAMM system described in [9], which predicts the signals exhibit no degradation despite being transmitted simultaneously. We demonstrate successful operation and provide results from our experiment that we compare with an ideal system. A video of our experiment is available as supplementary material relating to this paper. Our demonstration shows the potential for providing ultra-spectrally efficienct train-to-ground connectivity by multiplexing many data streams onto the same radio resource using carefully selected pre-coding, i.e. LAMM. With radio spectrum being scarce, this appears an extremely attractive option.

The sections in the remainder of this paper are as follows. Section 2 describes the LAMM concept in more detail. Section 3 describes the experimental system. The methodology is described in §4. Analytical results are shown in §5 and measured results in §6. Discussion is provided in §7 followed by conclusions in §8.

## 2. LAMM concept and experimental system

For the work reported in this paper, we use a 2 × 2 LAMM system comprising two antennas forming an array on the ground side of the communications link and the same for the train side. This has been used to create two LAMM modes, i.e. *l* = 0 and *l* =  + 1. These modes are the left-hand and middle modes shown in figure 1 in the ideal continuous form. For the *l* = +1 mode, phase linearly increases between 0 and 2*π* radians along the *x*-axis, i.e. along the railway track, which would then repeat; while for *l* = 0 the phase remains at zero. For each mode, the antenna has a total length of *D* and the phase varies with distance *d* along the range. For higher modes, the rate of phase change increases for non-zero values of *l*. The amplitude pattern will be determined by the antennas used, and ideally a flat amplitude profile over the range *D* is required to preserve the linear phase profile. Using figure 1, it can be inferred that for mode *l*, the relation between the phase *ψ* required at the transmitter and receiver at a given distance *d* along the plane that has total length *D* is as follows:

2.1 $$\psi_{\text{Tx}}(d)\;=\;l\frac{d}{D}2\pi \;\psi_{\text{Rx}}(d)=-l\frac{d}{D}2\pi .$$

Note that when the transmit and receive modes are matched, then *ψ*_Tx_ is the conjugate phase angle of *ψ*_Rx_ and when they are not, then this is not maintained. To demonstrate the multiplexing between the two available modes, a comparator function, *O*(*d*) can be applied as follows:

2.2 $$O(d)=\int_{0}^{D}{\text{e}}^{\text{j}\psi_{\text{Tx}}(d)}{\text{e}}^{\text{j}\psi_{\text{Rx}}(d)}dd,$$

and *O*(*d*) = 1 when the transmit and receive modes match, while if *l* = ±1 is transmitted and *l* = 0 is at the receiver (or vice versa) then *O*(*d*) = 0. The same happens where *l* = +1 is transmitted and *l* = −1 is at the receiver and vice versa. This, therefore, demonstrates the multiplexing capability of LAMM with the first three possible modes and can similarly work for |*l*| > 1.

A LAM mode may be created in discrete form by means of a linear array of two antenna elements where *N* = 2 as follows, where element *n* is at a discrete displacement point *d_n_*:

2.3*a* $$\psi_{\text{Tx}}(n)=l\frac{d_{n}}{D}2\pi =l\frac{n}{N}2\pi$$

and

2.3*b* $$\psi_{\text{Rx}}(n)=-l\frac{d_{n}}{D}2\pi =-l\frac{n}{N}2\pi .$$

**Figure 2.**
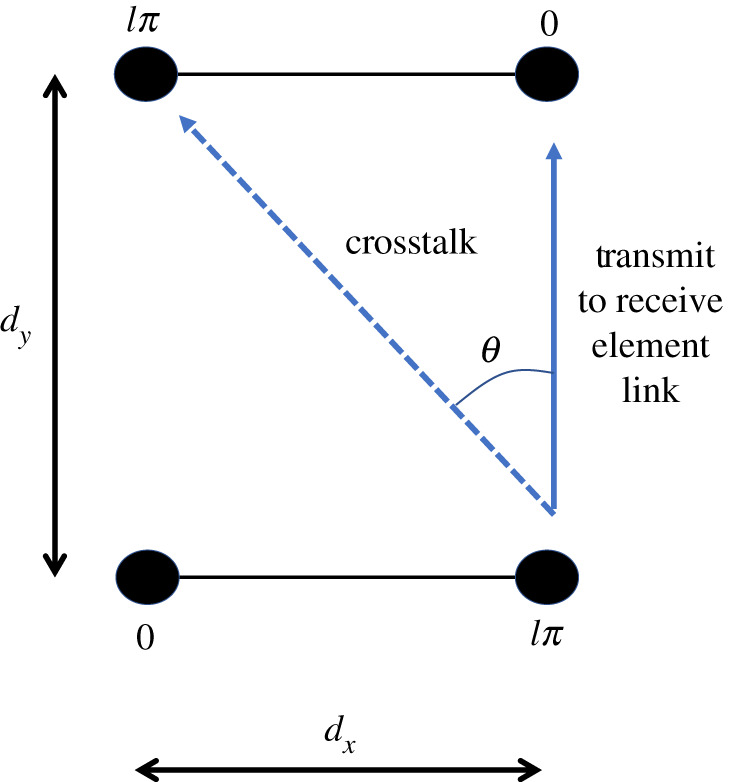
Illustration of the set-up for detection of LAM modes *l* = ±1 for an *N* = 2 linear array. (Online version in colour.)

In discrete form where *N* = 2, *l* = ±1 are degenerative modes such that only one of them can be used. However, *l* = ±1 is still orthogonal to *l* = 0, so a LAMM system with *N* = 2 has two orthogonal modes. Figure 2 shows a 2 × 2 LAMM system where the line of sight has distance *d_y_* from the transmitter to the receiver and the elements are separated in the *x-*direction (the same as the direction of travel) with separation *d_x_* between elements, which is the case for the experimental system described later. Ideally, a link is formed between only the transmit and receive antenna elements that are opposite each other, with no signal going to other adjacent receive elements as this would cause interference. However, in practice there would be some leakage due to the beamwidth of the elements' radiation patterns causing crosstalk, as indicated in figure 2. It is essential to ensure the topology is correctly set in order to have a viable link by carefully selecting the geometry determined by *d_x_* and *d_y_*, either to form a weak crosstalk link or a phase cancellation between the modes [10]. It is also determined by the level of leakage between feeder cables and devices used to create the required phase profile for the LAM modes. As with any link, the level of tolerable crosstalk is determined by the modulation and coding scheme, path loss of the wanted and unwanted signals, antenna radiation patterns, receiver noise and target bit error rate. While the above description relates to a static scenario of aligned antennas, in practice the train will move, and the antennas will go through cycles of being aligned and misaligned causing the signal-to-interference-plus-noise ratio (SINR) of each of the two modes to vary cyclically. To avoid interference occurring when the receiver is offset from the transmitter, pre-coding (i.e. LAMM) can be applied such that interference cancellation occurs while antennas are misaligned. For the *l* = +1 mode, this requires 180° phasing to be applied.

In this study, four patch antennas have been used at 2.35 GHz where the amplitude follows a cosine profile with a 3 dB beamwidth of 80° and all antennas are orientated with a vertical linearly polarized signal. In order to apply the LAM modes as shown in figure 2, 180° hybrid couplers are used at the transmitter and receiver whereby the sum combiner ports (Σ) can be used for the *l* = 0 mode and the difference combiner ports (Δ) for the *l* = +1 mode. The hybrid couplers could be removed or replaced in order to compare the transmission link with and without LAM pre-coding. Therefore, by selecting a low enough ratio of *d_x_*/*d_y_* it can create a substantial enough crosstalk without coding while applying the LAM pre-coding would counteract this. In this study a *d_x_*/*d_y_* ratio of 0.55 was chosen such that with the LAM pre-coding ([10], fig. 7b) it will provide a maximum phase cancellation between the modes to maximize data throughput. At the same time the spacing is not too narrow where it would be considered as a beamforming rich channel.

**Figure 3.**
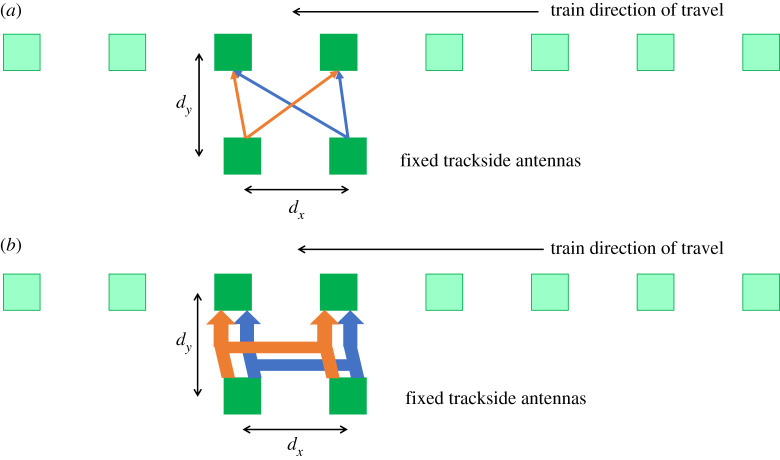
Illustration of the test concept for a 2 × 2 configuration used in a full deployment on a train to trackside scenario where (*a*) no LAMM is applied causing crosstalk and (*b*) two sufficiently orthogonal links are jointly formed with LAMM applied to minimize crosstalk. (Online version in colour.)

In the experimental set-up only a 2 × 2 link need be analysed to compare the link quality with and without LAMM as the antenna elements on board the train are offset from the trackside. Figure 3 illustrates a scenario where such antennas would be implemented in full on a train or moving platform. The light shaded antennas are shown as the ones not in use at the time and will activate as they come to face one of the two trackside antennas. By observing figure 3*a* directly it can be seen that without LAMM there is a direct crosstalk interference between the two active links due to no pre-coding being applied on the antennas, while with LAMM applied in figure 3*b*, it forms near orthogonal links with negligible enough crosstalk between the two modes formed as the active antennas pass each other. Finally, it can be noted that as the antenna element separation *d_x_* increases substantially, this would substantially reduce the crosstalk without LAMM applied, but this would then limit the number of modes that could be used in practice. Therefore, the separation can be brought closer with such pre-coding applied to enable the use of more modes.

## 3. Experimental LAMM system

**Figure 4.**
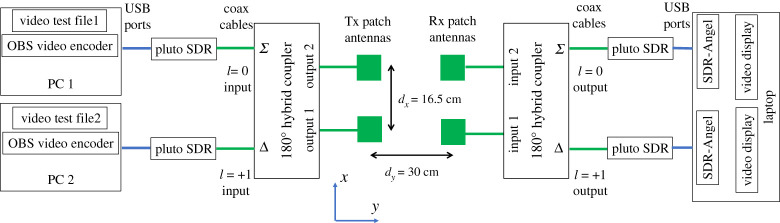
LAMM transmitter to receiver block diagram. Hybrid couplers are removed at the transmitter and receiver when testing with no coding. The Tx patch antennas slide in the *x*-direction. (Online version in colour.)

Figure 4 shows the block diagrams of the LAMM transmitter and receiver, respectively. The blocks are further specified in table 1. Furthermore, our system consists of two vertically polarized pairs of rectangular patch antennas, where the antennas have a 3 dB beamwidth of 80° and a centre frequency of 2.35 GHz. They are configured such that *d_x_* = 16.5 cm, and *d_y_* = 30 cm, hence $d_{x}/d_{y}=\;0.55$. This ensures that sufficient crosstalk is generated to show the benefit of applying LAMM to overcome it. The patch antenna dimension and operating frequency means that far field transition takes place at around 2 cm, and therefore the minimum link distance *d_y_* = 30 cm is well in the far field. While the dimensions of *d_x_* and *d_y_* are not representative of a realistic case such as a trackside to train scenario, as the antennas are operating in the far field the same results would be observed with larger dimensions but maintaining the same ratio, thus the findings in this work can be scaled to a real deployment. A 180° phase shift is required between the antennas in order to construct LAM modes of *l* = 0 and +1. This has been implemented by means of a 180° hybrid coupler each at the transmit and receive ends, as shown in figure 4, such that the sum (Σ) and difference (Δ) inputs and outputs correspond to *l* = 0 and +1, respectively. The two hybrid couplers are identical and when connected back to back, they were measured to have an isolation of 23 dB between the Σ–Σ and Σ–Δ (or Δ–Δ and Δ–Σ) links. This is equal to the achievable signal-to-interference-ratio (SIR) and, therefore, maximum SINR and hence the achievable spectral efficiency, determined by means of Shannon's channel capacity theorem [16] to be 18.6 bits s Hz^−1^. This SINR and subsequent capacity will inevitably be lower due to the addition of Gaussian noise and a weaker isolation with the antennas in use.

**Table 1.** LAMM system specification.

block	description	notes
PCs	Windows10 OS	PC1 and PC2 at the Tx, one laptop at the Rx
	USB2 ports	
	clock speed >2 GHz	
	4 GB RAM	
video encoder	OBS video-encoding software	installed on each Tx PC^1^
	H264 MPEG4 video codec	
	696 × 392 pixels	
	256 kb s^−1^ data rate	
	333 kb s^−1^ symbol rate	
transmitters	Analog Devices ADALM-PLUTO	two transmitters, in order that each one can stream the video down each mode^2,3^
	SDRs	
	modulator and up-convertor	
	F5OEO DVB firmware	
	DVB-S2	
	QPSK 2/3 rate LDPC/BCH FEC	
	2.35 GHz carrier frequency	
	transmit power −2 dBm	
receivers	Analog Devices ADALM-PLUTO	two receivers, in order that each one can receive the two streamed transmissions down each mode^3^
	SDRs	
	default firmware	
	down-convertor function	
	2.35 GHz carrier frequency	
	noise figure 3 dB	
	unfiltered noise floor −50 dBm	
	20 MHz bandwidth	
demodulation and decoding software	SDR-Angel software configured according to Tx waveform and video encoder	installed on each Rx PC^4^
hybrid coupler	Qotana DBHB1802000400 2–4 GHz	x1 for Tx, x1 for Rx^5^
	180° hybrid coupler, 22 dB isolation	
antennas	80° 3 dB beamwidth patch	
	vertical polarization	
	directivity ∼6 dB	
	maximum return loss at 2.35 GHz	
LAM link geometry	*d_x_* = 16.5 cm, *d_y_* = 30 cm	

With reference to the transmitter in figure 4, two personal computers (PCs) are used to encode the two digital video streams. The video content can be either streamed from connected ‘web cams’, or text/graphics provided from the PC. In this case a ‘test card’ stating the channel number was used. Channel 1 was red text and at the bottom of the image, and channel 2 was green text and at the top of the image. The two video streams were each connected to a SDR, which modulated the signals according to the DVB-S2 signal specification (table 1) and then up-converted them to 2.35 GHz at a power of −2 dBm. These signals then connected to the two inputs of the hybrid coupler by means of 1 m coaxial cables with less than 0.3 dB loss. The output ports of the hybrid have a 0° and 180° phase shift between them for *l* = 0 and *l* = +1 modes, respectively, which are connected to the two patch antennas.

The receiver block diagram is depicted in the receiver of figure 4 and consists of two patch antennas, which are connected to the 180° hybrid combiner to receive the *l* = 0 and *l* = +1 modes. The two outputs of the combiner each connect to two SDRs, which down-convert the signals and outputs them to the PC via USB connections. The PC runs two instances of demodulation/decoding software called SDR-Angel; one instance for each channel. The software configures the SDRs to the wanted frequency and gain, etc. and then demodulates and decodes the received signals. It also decodes and displays the resulting video image. An indication of the received signal power is also provided. It should be noted that the Tx patch antennas slide in the *x*-direction when taking measurements. The same test is carried out without LAMM coding by removing the hybrid couplers and connecting the coax cables directly to the antennas.

**Figure 5.**
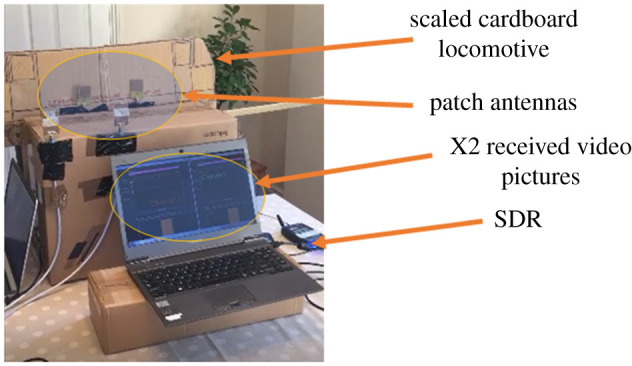
Complete 2 × 2 experimental LAMM system. (Online version in colour.)

Figure 5 shows a photograph of the system. The four patch antennas are shown along with one of the SDRs. The PC used to demodulate/decode the signals is also shown, along with the two received video pictures, as indicated in figure 5. The figure also shows the test rig, which consists of a cardboard cut-out of a scaled down railway locomotive that has a pair of antennas attached to it. The patch antennas have their own ground plane so it is not necessary to use a metallic cutting for this purpose. This is mounted on a cardboard box and can be slid laterally, guided by the groove cut along the length of the box, which acts like a track. The second pair of antennas are mounted on the edge of the box. The set-up has been used for the subsequent measurements and was located away from any immediate objects to minimize multi-path effects due to the environment. The closest wall was 1.5 m away and would not have caused reflections in the direction of the receive antennas.

## 4. Methodology

Measured and analytical results were obtained using the experimental LAMM system and the methods applied in post-processing the data to obtain comparative results with and without LAMM pre-coding are described in this section.

Received power measurements were taken and compared with those expected in an ideal scenario. Received power measured at each receiver also enabled the SINR and spectral efficiency to be determined. The received video images were also observed to ascertain if they were correctly received and decoded. If the link was not operating as expected, an image would either not be received, or the image from the other channel would be received as interference and subsequently displayed.

Prior to taking any measurements, the transmit power for each channel was selected such that the receivers remained linear (no saturation) and that the received video images were displaying without distortion with the antennas in alignment and with LAMM coding. The transmit power was then reduced such that it was the minimum required to sustain distortion-free images. This meant that the link margin was such that the forward error correction (FEC) was effective, which required an SINR of at least 3.1 dB for QPSK modulation and 2/3 rate FEC.^6^ The transmit power between each channel was also balanced such that, with the antennas in alignment and one channel enabled at a time, the received power on each channel was the same. This choice of transmit power meant that any reduction in received SINR, which results from the presence of interference from the other channel, would cause the link to become inoperational. Therefore, the two video streams are not separated and the stronger of the two channels appears on both monitors at the receiver, making the dysfunctional link visible.

Single channel measurements were first completed without LAMM coding, i.e. with the hybrid couplers removed at the transmitter and receiver. A single transmitter was enabled, then the procedure was repeated for the second transmitter. With one transmitter enabled, the received signal power was measured at each receiver and for each positional increment of the two antennas mounted on the locomotive as it was moved along a linear path. Increments occurred in 2 cm steps up to a maximum displacement of 14 cm from the starting location. The quality of the received image was also noted for each measurement position along with the received signal power at each receiver.

Dual channel measurements occurred in the same way as the single channel measurements except that both the transmitters were enabled. Following this, the hybrid couplers were inserted at the transmitter and receiver and the measurements repeated.

In addition to the measured results, an idealized system was also developed analytically to enable comparison with the measured data. As with the above, the analysis considered the received signal at the receive antennas for when the 180° phase is enabled and removed from the system as the antennas are incremented in the *x*-direction along a 14 cm trajectory in 2 cm intervals. An ideal antenna with a uniform amplitude and phase response between ±80°, i.e. a ‘top hat’ response, has been analysed, as well as a cosine amplitude response that synthesizes the patch antenna. The analytical results were determined by computing the geometric distance and angle between the antenna pairs at each position along the trajectory. This distance *d* determined the resulting phase, and the angle *θ* in radians illustrated in figure 2 determined the attenuation *A* to the channel coefficient resulting from the transmit and receive antenna patterns at that angle. For the uniform antenna this made no difference, but for the patch antennas, this was determined by the cosine model given by equation (4.1). The factor of 1.13, found by trial and error, in equation (4.1) results in a 3 dB beamwidth of 80° to match the antennas used for the measurements. The angular phase response of the antennas was not required as the effect from the transmit antenna is cancelled out by the facing receive antenna.

4.1 A(θ)=cos(1.13θ).

The amplitude and phase of the signals arriving from each of the transmit antennas at each of the received antennas was then summed vectorially, giving a resultant amplitude and phase. For the LAMM coding, an additional 180° phase was added at the transmitter to channel 2, and the same at the receiver. This synthesizes the action of the two hybrids.

The results are split between those obtained by analysis and those from the experimental system. They are displayed as a function of lateral displacement of the mobile platform with respect to the starting location, with and without LAMM coding to show the impact of LAMM. These results are as a function of SIR (dB) defined in (4.2*a*) and (4.2*b*) for channel coefficients *h*_ΣΣ_ and *h*_ΔΔ_ with crosstalk coefficients *h*_ΣΔ_ and *h*_ΔΣ_ in the case of when LAMM coding (i.e. using the hybrid couplers in figure 4) is applied. SIR is also defined in (4.3*a*) and (4.3*b*) for the case without LAMM coding and the hybrid couplers removed, such that the channel coefficients between transmit and receive antenna pairs 1 and 2 given as *h*_11_ and *h*_22_ with crosstalk coefficients *h*_12_ and *h*_21_. It is, therefore, expected that the LAMM coding will give a higher SIR due to having a lower crosstalk. The results are also expressed in terms of spectral efficiency (*ξ*) (bits s^−1^ Hz^−1^) using the Hartley–Shannon channel capacity formula [16] and defined by expressions (4.4*a*) and (4.4*b*). Note here that noise is excluded and so the SIR will be used as the maximum possible SINR achievable. For the scope of this work, negligible noise compared with the interference power and Gaussian interference power distribution are assumed [17], where detailed analysis of this is beyond the scope of this work.

4.2*a* $$\begin{array}{rl} & \text{SI}{\text{R}}_{\text{channel }1|\text{LAMM}}=10\;\text{lo}{\text{g}}_{10}\left(\frac{h_{\Sigma \Sigma }}{h_{\Delta \Sigma }}\right), \end{array}$$

4.2*b* $$\begin{array}{rl} & \text{SI}{\text{R}}_{\text{channel }2|\text{LAMM}}=10\;\text{lo}{\text{g}}_{10}\left(\frac{h_{\Delta \Delta }}{h_{\Sigma \Delta }}\right), \end{array}$$

4.3*a* $$\begin{array}{r} \text{SI}{\text{R}}_{\text{channel }1|\text{no LAMM}}=10\text{lo}{\text{g}}_{10}\left(\frac{h_{11}}{h_{21}}\right), \end{array}$$

4.3*b* $$\begin{array}{r} \text{SI}{\text{R}}_{\text{channel }2|\text{no LAMM}}=10\text{lo}{\text{g}}_{10}\left(\frac{h_{22}}{h_{12}}\right), \end{array}$$

4.4*a* $$\begin{array}{r} \xi_{\text{channel }1}=\text{lo}{\text{g}}_{2}(1+\text{SI}{\text{R}}_{\text{channel }\;\;1}) \end{array}$$

4.4*b* $$\begin{array}{r} \;\text{and}\;\xi_{\text{channel }2}=\text{lo}{\text{g}}_{2}(1+\text{SI}{\text{R}}_{\text{channel }\;2}). \end{array}$$

The SIR and spectral efficiency are analysed theoretically for two cases: (i) where the effect of antenna patterns of the patch antennas are excluded and, therefore, *A*(*θ*) = 1 for all angles defined as a ‘top hat’ antennas scenario; (ii) where the effect of the antenna patterns of the patch antennas are included and, therefore, *A*(*θ*) as defined by (4.1) is subsequently used and can be considered as ‘cosine’ antennas. The subsequent channel coefficients can be computed as well as the LAMM pre-coded channels, as detailed in [10], assuming free space between the transmit and receive antennas.

## 5. Analytical results

**Figure 6.**
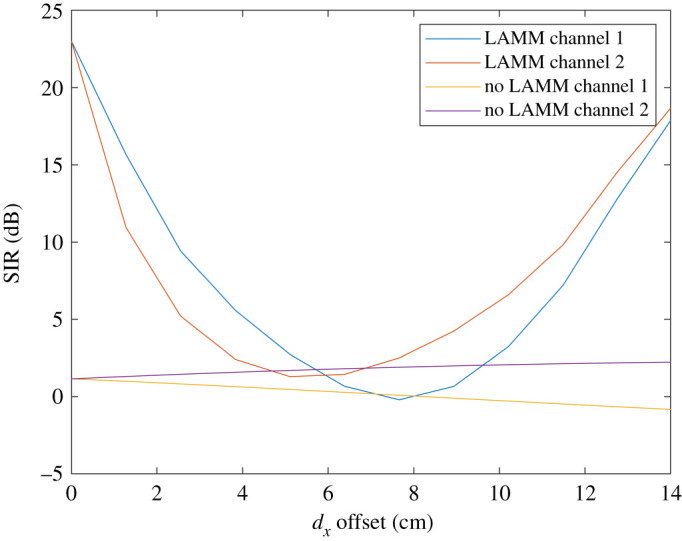
Analytical results for SIR versus lateral offset for when ‘top hat’ antennas are used. (Online version in colour.)

**Figure 7.**
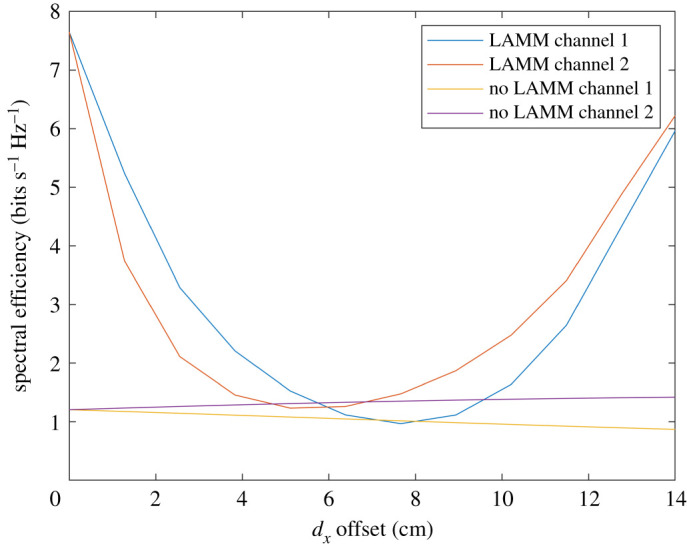
Analytical results for spectral efficiency for when ‘top hat’ antennas are used. (Online version in colour.)

Theoretical results are compared in this section for SIR and spectral efficiency with and without LAMM coding. Figures 6 and 7 show performance obtained by means of analysis for when ‘top hat’ antennas are used. Figure 6 shows the SIR at each of the two receive antennas and for when uncoded and LAMM coding is used and it increases substantially. Variations of the SIR result from the path length (and subsequent path loss) varying as the platform moves along the 14 cm path. Figure 6 shows improvement in SIR for much of the path for when LAMM coding is used, except in the region close to 6–8 cm offset, such that the phase of the channel coefficients do not enable the LAMM coding to suppress the crosstalk, as is achieved at no offset and moving towards 14 cm offset. Similarly, figure 7 shows the corresponding spectral efficiency, with a maximum spectral efficiency of 1.4 bits s^−1^ Hz^−1^ for the uncoded case, and 7.5 bits s^−1^ Hz^−1^ for the LAMM-coded case occurring when the arrays are aligned, i.e. 0 cm offset.

**Figure 8.**
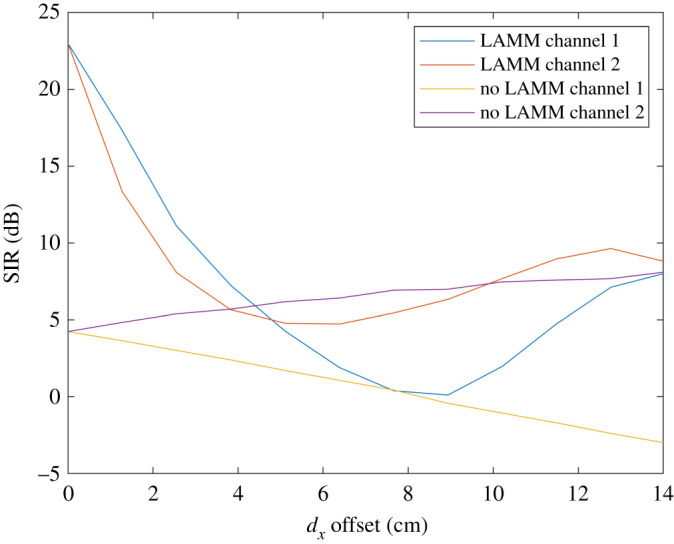
Analytical results for SIR versus lateral offset for when ‘cosine’ antennas are used. (Online version in colour.)

**Figure 9.**
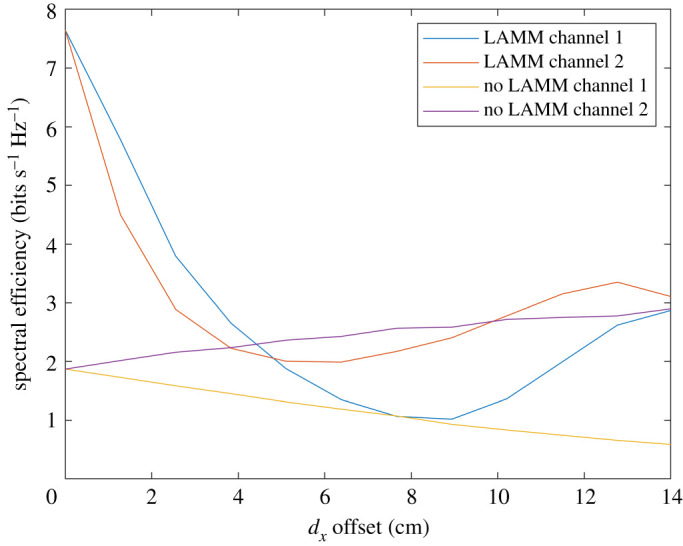
Analytical results for spectral efficiency for when ‘cosine’ antennas are used. (Online version in colour.)

Figures 8 and 9 show the results obtained by analysis for when ‘cosine’ antennas are used. Figure 8 shows the SIR at each of the two receive antennas with and without LAMM coding. Compared with the ‘top hat’ case, it is evident that the antenna patterns prevent the capacity reaching a peak again at 14 cm offset as the pattern will weaken the channel between the transmit and receive antenna pairs 1 and 2 at wide angle, *θ*, while the crosstalk will be more substantial, corresponding to a lower *θ*. Nonetheless figure 8 shows improvement in SIR for smaller offset when LAMM coding is used. Similarly, figure 9 shows the corresponding spectral efficiency.

## 6. Measured results

**Figure 10.**
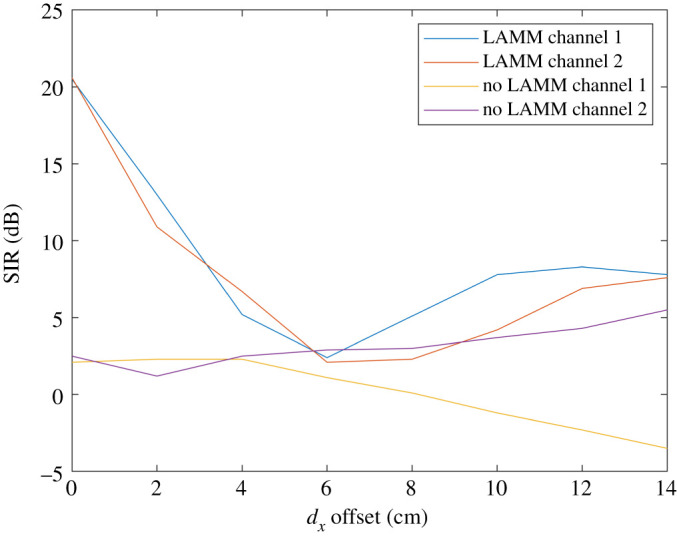
SIR derived from measured data. (Online version in colour.)

Figure 10 shows measured SIR levels as a function of lateral displacement of the mobile platform. SIR values (dB) have been derived from the measured received signal strength indicator (RSSI) data measured in dBm for every 2 cm offset both with and without the hybrid couplers present, such that expressions (6.1*a*) and (6.1*b*) correspond to the case with LAMM coding and (6.2*a*) and (6.2*b*) correspond to the case without.

6.1*a* $$\begin{array}{r} \text{SI}{\text{R}}_{\text{channel }1|\text{LAMM}}=\text{RSS}{\text{I}}_{\Sigma \Sigma }-\text{RSS}{\text{I}}_{\Sigma \Delta }, \end{array}$$

6.1*b* $$\begin{array}{r} \text{SI}{\text{R}}_{\text{channel }\;2|\text{LAMM}}=\text{RSS}{\text{I}}_{\Delta \Delta }-\text{RSS}{\text{I}}_{\Delta \Sigma }, \end{array}$$

6.2*a* $$\begin{array}{r} \text{SI}{\text{R}}_{\text{channel}\;\;1|\text{no LAMM}}=\text{RSS}{\text{I}}_{11}-\text{RSS}{\text{I}}_{12} \end{array}$$

6.2*b* $$\begin{array}{r} \;\text{and}\;\text{SI}{\text{R}}_{\text{channel}\;2|\text{no LAMM}}=\text{RSS}{\text{I}}_{22}-\text{RSS}{\text{I}}_{21}. \end{array}$$

**Figure 11.**
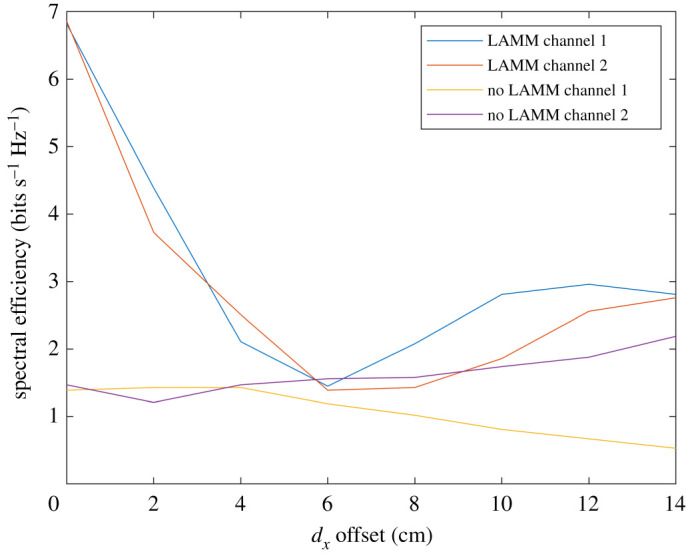
Spectral efficiency derived from measured data. (Online version in colour.)

The resulting curves are shown in figure 10, which depict a distinct improvement in SIR for the LAMM-coded case when compared with the uncoded case. According to the DVB-S2 SINR requirements described in (see footnote 1), the uncoded link does not render an SIR high enough to operate satisfactorily. This was observed in practice since the level of interference meant that the same image was observed on both channels. This was not the case when LAMM coding was used and even the lowest SIR of approximately 3.1 dB was still sufficient to sustain the video link. Figure 11 depicts the corresponding spectral efficiency, showing a consistently high capacity for when LAMM is used compared with when it is not. The results from the measured system show good correspondence compared with the simulated results, which is a good indicator that the phase and attenuation impairments in the fabricated patch antenna patterns, which are unaccounted for in the theoretical analysis, exhibit minimal effect. A short video showing this experiment is available as supplementary material relating to this paper.

## 7. Discussion

**Figure 12.**
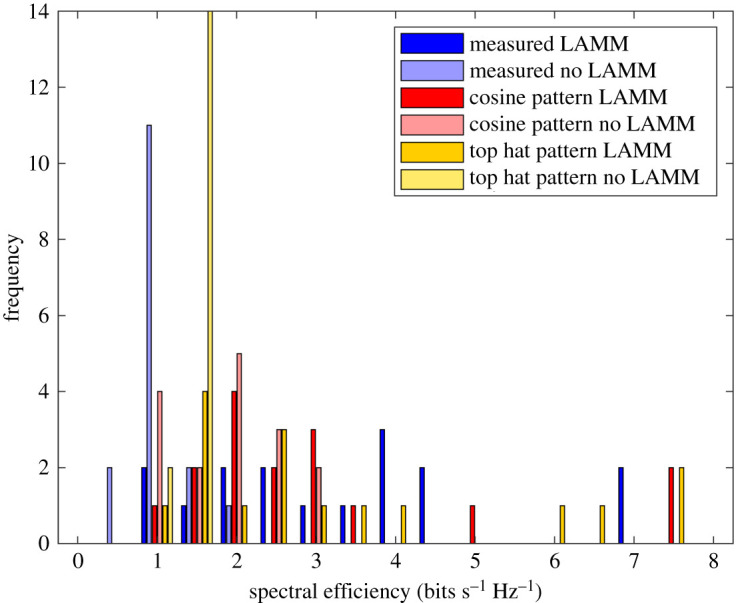
Histogram of spectral efficiency for LAMM-coded and uncoded links and for ‘top hat’ antenna, ‘cos’ antenna and measured scenarios. (Online version in colour.)

Figure 12 shows a histogram of achieved spectral efficiency for LAMM-coded and uncoded scenarios for when ‘top hat’ and ‘cosine’ antennas are used, as well as for the measured results. This shows that for all scenarios LAMM coding provides a spectrum efficiency improvement when compared with the uncoded scenario. This is further corroborated in table 2, which summarizes the spectral efficiency results in terms of minimum, maximum and mean spectrum efficiencies.

**Table 2.** Minimum, maximum and mean achieved spectral efficiencies for LAMM-coded and uncoded links and for ‘top hat’ antenna, ‘cosine’ antenna and measured scenarios.

spectral efficiency (bits s Hz^−1^)	min	max	mean
no LAMM top hat pattern	0.95	1.5	1.19
LAMM top hat pattern	1	7.5	3.2
no LAMM cosine pattern	0.5	2.9	1.7
LAMM cosine pattern	1	8	3
no LAMM measured	0.53	2.19	0.8
LAMM measured	1.56	7	3.1

**Figure 13.**
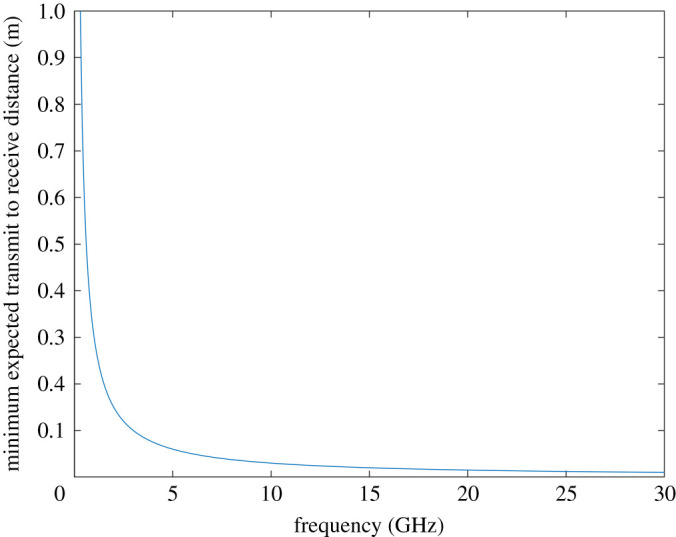
Plot of minimum expected transmit to receive distance to meet far field criteria versus frequency. (Online versionin colour.)

Regarding variations in the measured results compared with the theoretical case, we suggest that selecting lower operating frequencies would reduce the impact as the wavelength becomes longer compared with geometric variations and hence would lower the impact. Furthermore, the design of the antennas to resemble the flat antenna pattern with constant phase patterns is also important in sustaining phase stability. The velocity of the train or moving vessel will have little impact on the phase variation in such a channel as the Doppler shift for a link transverse to the direction of propagation is minimal and reduced even further with low frequency. The lowest operating frequency is, however, bounded by ensuring far field operation and practical antenna size implications. It would be expected that an antenna element would typically have a maximum dimension of half a wavelength or less while a transmit to receive distance in the context of ground to train would be at least 1 m. This would correspond to meaning that the Rayleigh distance would be a half wavelength or less and, therefore, that the minimum required transmit to receive distance equal to *d_y_* would be twice this value and, therefore, one wavelength. It can be seen by plotting this distance requirement versus frequency in figure 13 that above 1 GHz, this criterion is sufficiently met, which covers the likely available spectrum below 6 GHz.

In a real-world system, there would be reflections between train and ground, e.g. rails, sleepers, ballast (if antennas are on the ground). Hence this would contribute to the reduction in Rician K-factor, where the resulting multi-path may have a negative effect. However, according to simulation results provided in [10], which show spectral efficiency for a 2 × 2 LAM link with Rician K-factor of 20 and 6, the spectral efficiency is shown to reduce only from 5 bits s Hz^−1^ to 4 bits s Hz^−1^, with the exact performance being sensitive to *d_x_*/*d_y_* and showing variations of approximately ±10%. This indicates that the performance is sensitive to the link geometry.

## 8. Conclusions

Experimental evaluation of a LAMM radio link for moving platforms has been described and evaluated in this paper. We have presented results obtained from a scale experimental system comprising a 2 × 2 antenna system operating at 2.35 GHz with the link transmitting two independent digital video streams. Linear motion is introduced to demonstrate the translation-invariance of the technique as the platform progresses along a lateral route. The measured data have been compared with those obtained from an analytical theory-based model and show that crosstalk between the two channels is at levels low enough to consistently support the video streams without interruption. Specifically, our results show that spectral efficiency is consistently higher when LAMM coding is enabled compared with an uncoded channel, while any practical phase and amplitude impairments caused by fabricated antennas have a minimal effect.

Further work in developing the LAMM concept is suggested to focus on: exploring planar two-dimensional LAMM as described in [10], extending the system to support longer lateral movements, more modes, novel antenna designs and implementing dual polarization to provide additional minimization of crosstalk.

## Footnotes

1
https://obsproject.com/

2
https://www.analog.com/en/design-center/evaluation-hardware-and-software/evaluation-boards-kits/adalm-pluto.html

3
http://www.vivadatv.org/viewtopic.php?f=87&t=685

4
https://github.com/f4exb/sdrangel

5
http://www.qotana.com/pdf/id/149/mod/Hybrid

6
http://www.satbroadcasts.com/news,81,Minimum_carrier_to_noise_ratio_values_CNR,_CN_for_DVB_S2_system.html

## Data accessibility

Measured data used to produce the measured results in §§6 and 7 are available from the DOI given below. https://doi.org/10.5061/dryad.r4xgxd2c4.

The data are provided in the electronic supplementary material [18].
